# Diagnostic accuracy of cytology and urine methylation test in patients with non-muscle invasive bladder cancer: a systematic review and meta-analysis

**DOI:** 10.3389/fonc.2024.1412346

**Published:** 2024-09-03

**Authors:** Zhuoyue Yao, Tao Wang, Jingpeng Liu, Zhongbao Zhou, Yong Zhang

**Affiliations:** Department of Urology, Beijing Tiantan Hospital, Capital Medical University, Beijing, China

**Keywords:** NMIBC, urine methylation test, receiver operating characteristic, meta-analysis, diagnose

## Abstract

**Background:**

Multiple clinical studies have demonstrated the numerous advantages of urine methylation test over cytology for monitoring patients with non-muscle invasive bladder cancer (NMIBC) following surgery. This research aims to provide a systematic review and meta-analysis to evaluate the efficacy and limits of urine methylation test in the clinical management of NMIBC.

**Methods:**

This research was carried out by conducting a comprehensive search of clinical trials comparing cytology and urine methylation test for NMIBC follow-up using databases such as PubMed, Embase, Web of Science, and the Cochrane Library up until May 2023, including references from relevant articles. The study is registered on PROSPERO with ID CRD42023398969.

**Result:**

This study comprised six studies with a total of 1676 patients. The analysis revealed that the AUC of urine methylation test had a greater AUC than that of the cytology examination (0.89 vs 0.71). In post-operative follow-up of patients with NMIBC, the urine methylation test demonstrated a significant sensitivity (0.69 vs 0.52), but with lower specificity (0.87 vs 0.93) than cytology examination.

**Conclusion:**

The urine methylation test and cytology examination have both shown strong diagnostic performance in screening for NMIBC patients. However, urine methylation test outperforms cytology examination in terms of accuracy and sensitivity.

**Systematic review registration:**

PROSPERO, identifier CRD42023398969.

## Introduction

Bladder cancer (BC) is the sixth most prevalent malignancy and ninth leading cause of cancer death worldwide, with over 573,000 new cases and 213,000 deaths (1). According to GLOBOCAN 2020, cancer mortality rates are 9.5 per 100,000 males and 3.3 per 100,000 women, indicating that men have a nearly fourfold greater incidence than women. Smoking is the most important risk factor for BC, with a population attributable risk of approximately 50%. Epidemiology has indicated that while the incidence trend of BC has remained steady in males, from the 1990s to the 2020s, the incidence has grown with the rise of smoking prevalence in women (2). Since non-invasive tumors account for a considerable proportion of BC, early identification, timely intervention, and rigorous follow-up is critical to lowering the risk of recurrence and progression. The European Association of Urology (EAU) now recommends cystoscopy and urine cytology as the most frequently performed procedures for the diagnosis and follow-up of BC (3). Cystoscopy is an invasive test that might cause pain for BC patient as well as iatrogenic lesions and infections. Nevertheless, this technique remains significant in the diagnosis and monitoring of BC because to its remarkable specificity and sensitivity, particularly when performed by a trained surgeon (4). Urinary cytology, as the most often used non-invasive testing approach, nevertheless has limited accuracy, which is dependent on the expertise of pathologists (5, 6). Furthermore, the consistency and precision of the cytomorphological assessment may change throughout the therapy or development of BC (7).

The majority of newly diagnosed BC cases are non-muscle invasive bladder cancer (NMIBC), with most of these cases being limited to the mucosa (8).The recommended treatment options in the urology guidelines mainly include transurethral resection of bladder tumors, intravesical chemotherapy, and systemic chemotherapy for NMIBC (3). NMIBC, with its varied clinical histories, high risk of recurrence and propensity for advancement, need rigorous follow-up and continued monitoring after first therapy (9).

The significance of DNA methylation in the transition from NMIBC to invasive BC has been substantiated by scientific investigations (10). After detecting BC based on methylation profiles, researchers discovered that these markers are expected to have a more major role in the diagnosis and subsequent surveillance of NMIBC (11). Several studies have demonstrated the unique benefits of bladder methylation markers in diagnosing disease progression as compared to urine cytology during follow-up of individuals with NMIBC (12–14).

The urine methylation test has been shown to be clinically valid in multiple investigations. However, evidence-based medicine does not yet include a comparison of urine methylation test and urine cytology in follow-up patients with NMIBC. To assess their effectiveness and accuracy, we conducted a meta-analysis of the included studies, as required by evidence-based medicine, to guide subsequent research and clinical decision making.

## Materials and methods

### Search strategy

The systematic review was carried out in accordance with the requirements of the Preferred Reporting Items for Systematic Reviews and Meta-Analyses (PRISMA). The study protocol is registered on PROSPERO with ID CRD42023398969. We searched the PubMed, Embase, Web of Science, and Cochrane Library databases for published English-language studies evaluating the diagnostic value of urine methylation test for NMIBC up to May 2023. The search strategy incorporated Medical Subject Headings (MeSH) and equivalent terms.

### Inclusion criteria and exclusion criteria

We selected articles using the following inclusion criteria: (1) Pathological test findings were incorporated for BC classification; (2) Enrolled patients had been diagnosed and underwent initial treatment for NMIBC; (3) Patients must undergo both urinary cytology and urinary methylation test; (4) At least one of the following outcomes had to be assessed: diagnostic test accuracy expressed in terms of sensitivity (SE), specificity (SP), positive predictive value (PPV) and negative predictive value (NPV); (5) Published in English. The following were the exclusion criteria: (1) Case reports, letters, animal models, review articles, and meta-analyses; (2) Inadequate data, such as the number of patients based on the cut-off of urine methylation test, was not accessible.

### Study selection and quality assessment

We utilized a modified version of the QUADAS-2 tool to assess the methodological quality of the selected studies. To identify relevant studies, two researchers independently assessed the title, abstract, and full text. The following information were extracted and recorded: first author’s name, date and place of publication, sample size, male to female ratio, age, and type of study. Two researchers extracted the data, while a third researcher settled any discrepancies.

### Statistical analysis

All analyses were performed using the statistical software R (version 5.3.0, R Foundation for Statistical Computing, Vienna, Austria) in combination with the “mada” and “meta” packages. Statistical analysis was performed using a significance threshold of *p* < 0.05. The true positives (TP), false positives (FP), false negatives (FN), and true negatives (TN) were extracted from the selected studies. To evaluate the diagnostic effectiveness of urine methylation test, we performed a meta-analysis including sensitivity, specificity, diagnostic odds ratio, and patient manipulation characteristics. The chi-square test was performed to assess the heterogeneity of results. If I^2^ is less than 50%, a fixed-effect model is utilized because there is no detectable heterogeneity. Otherwise, a random effects model will be applied. To assess the methodological quality of the included articles, we employed the QUADAS-2 tool and data was analyzed using RevMan 5.2 software (15).

## Results

### Screening research process

**Figure 1:**
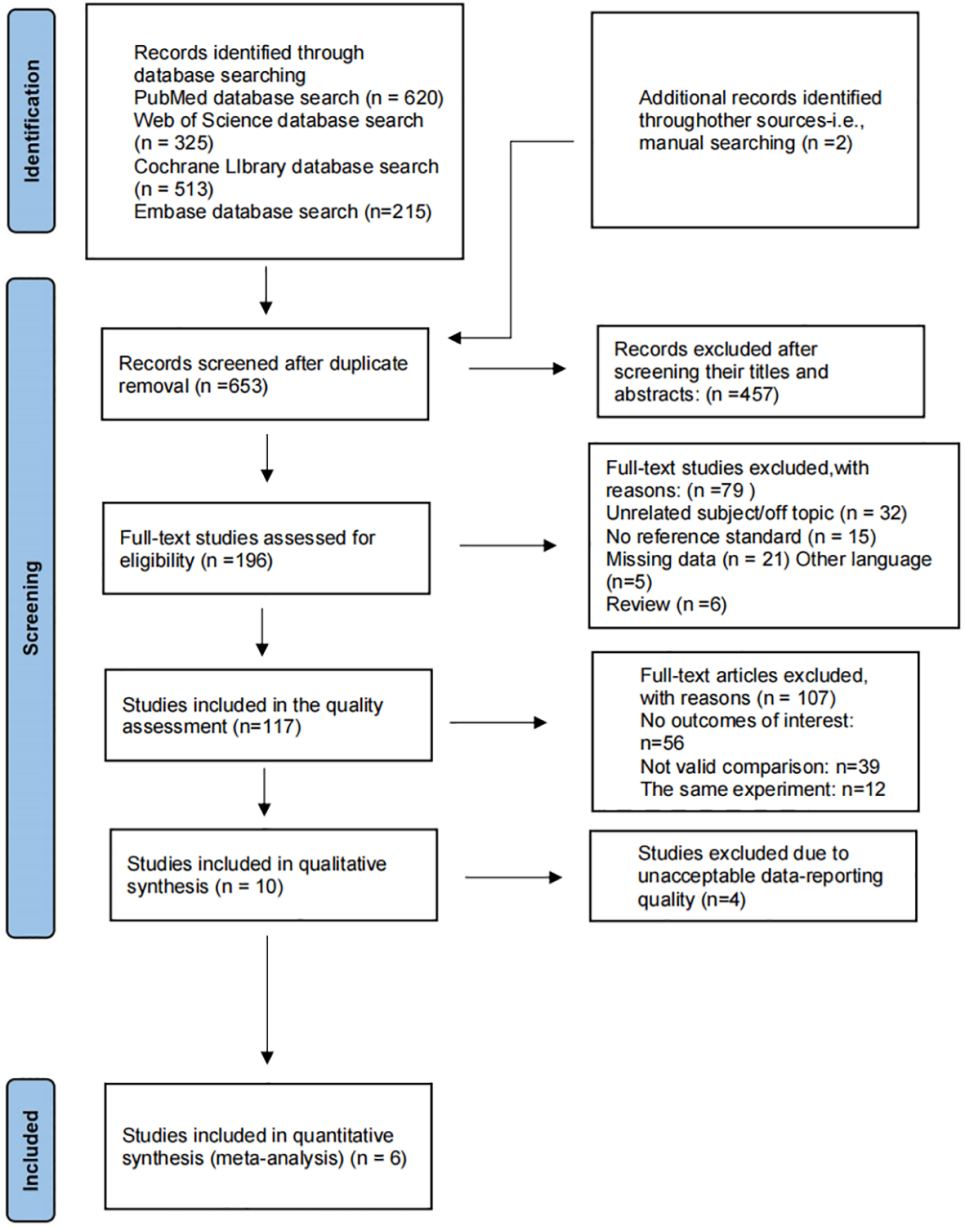
Flow diagram for systematic review.

**Table 1** presents the final consent search items based on the PIRD recommendations (**Table 1**) (16). We identified 620 records from PubMed, 215 from Embase, 325 from Web of Science, 513 from the Cochrane Library (**Figure 1**). The titles and abstracts of 653 records were screened after removing 1,020 duplicate records. Full texts for 196 studies were obtained. The quantitative analysis consists of ten articles. Four studies were removed because of poor quality. Finally, data is taken from 6 studies (17–22).

**Table 1:** Search strategy and documentation of keywords according to the PIRDS concept.

Population/Problem (P)	Index test (I)	Reference test (R)	Diagnostic and study type (D/S)
Non-Muscle-Invasive Bladder Neoplasms	Urine Methylation Test	valid*	Systemat*
NMIBC	Urine cytology	accuracy	Diagnos*
Non-Muscle-Invasive Bladder Cancer	Cytopathology	sensitivity	Detect*
Bladder Cancer, Non-Muscle-Invasive	Cytopathologies	specificity	Assessm*
Bladder Cancers, Non-Muscle-Invasive		SE	Study
Cancer, Non-Muscle-Invasive Bladder		SP	Studies
Cancers, Non-Muscle-Invasive Bladder		Positive predictive value	
Non-Muscle-Invasive Bladder Cancer		Negative predictive value	
		PPV	
		NPV	
		reproducib*	
		reliab*	
		threshold	
		cut-off	

* This keyword is a free word, which can be followed by any letter.

### Characteristics included in the study

We have generated a summary of authors, publication years, sample sizes and other information from the included articles (**Table 2**). We have compiled the threshold values for cytology and urine methylation test, together with their diagnostic significance (**Table 3**). The six studies involved 1,676 participants, and a standard cut-off was used consistently across all research. In all investigations, PCR detection of bladder tissue samples was carried out using a urine methylation test kit, and the data were processed using urine manipulation software to generate an EpiScore ranging from 0-100. A positive EpiScore is ≥60, whereas a negative score < 60. Cytology was assessed by pasteurized staining, and Paris Urine Cytology Reporting System was used to make the diagnosis. Patients ranged in age from 65.1 to 74, with 63.2 percent to 82.86 percent being male.

**Table 2:** Research background and design information of the included studies.

No.	Author	Year	Region	Types	Mean age	Male(%)	Sample size	Study design	Study population
1	Peña, K	2022	Spain	NMIB	NA	82.86	70	prospective	Single center
2	Ragonese, M	2022	Italy	NMIB	73.5	63.2	231	prospective	Single center
3	Pierconti,F	2022	Italy	NMIB	65.1	70.1	375	retrospective	Single center
4	Trenti, E	2020	Italy	NMIB	74	77.8	432	prospective	Single center
5	Territo A	2019	Spain	NMIB	74	81.1	215	prospective	Single center
6	Witjes, J	2018	Spain	NMIB	70.5	77.5	353	prospective	multicenter

**Table 3:** Parameters of diagnostic experiments included in the studies.

		Bladder EpiCheck test						cytology					
No.	Author	Cut off	TP	FP	FN	TN	Sensitivity (HG)	Cut off	TP	FP	FN	TN	Sensitivity (HG)
1	Peña, K	60	14	4	1	50	90.91	Paris System	18	6	2	50	85.71
2	Ragonese, M	60	58	20	15	151	91	Paris System	49	24	29	142	81
3	Pierconti,F	60	114	17	94	150	NA	Paris System	144	70	64	13	NA
4	Trenti, E	60	60	61	32	279	78.95	Paris System	25	3	67	337	47.37
5	Territo A	60	43	20	26	126	83.3	Paris System	23	1	46	145	66.7
6	Witjes, J	60	30	37	14	272	NA	Paris System	12	1	18	13	NA

TP, true positive; TN, true negative; FP, false positive; FN, false negative.

### Sensitivity and specificity

**Figure 2:**
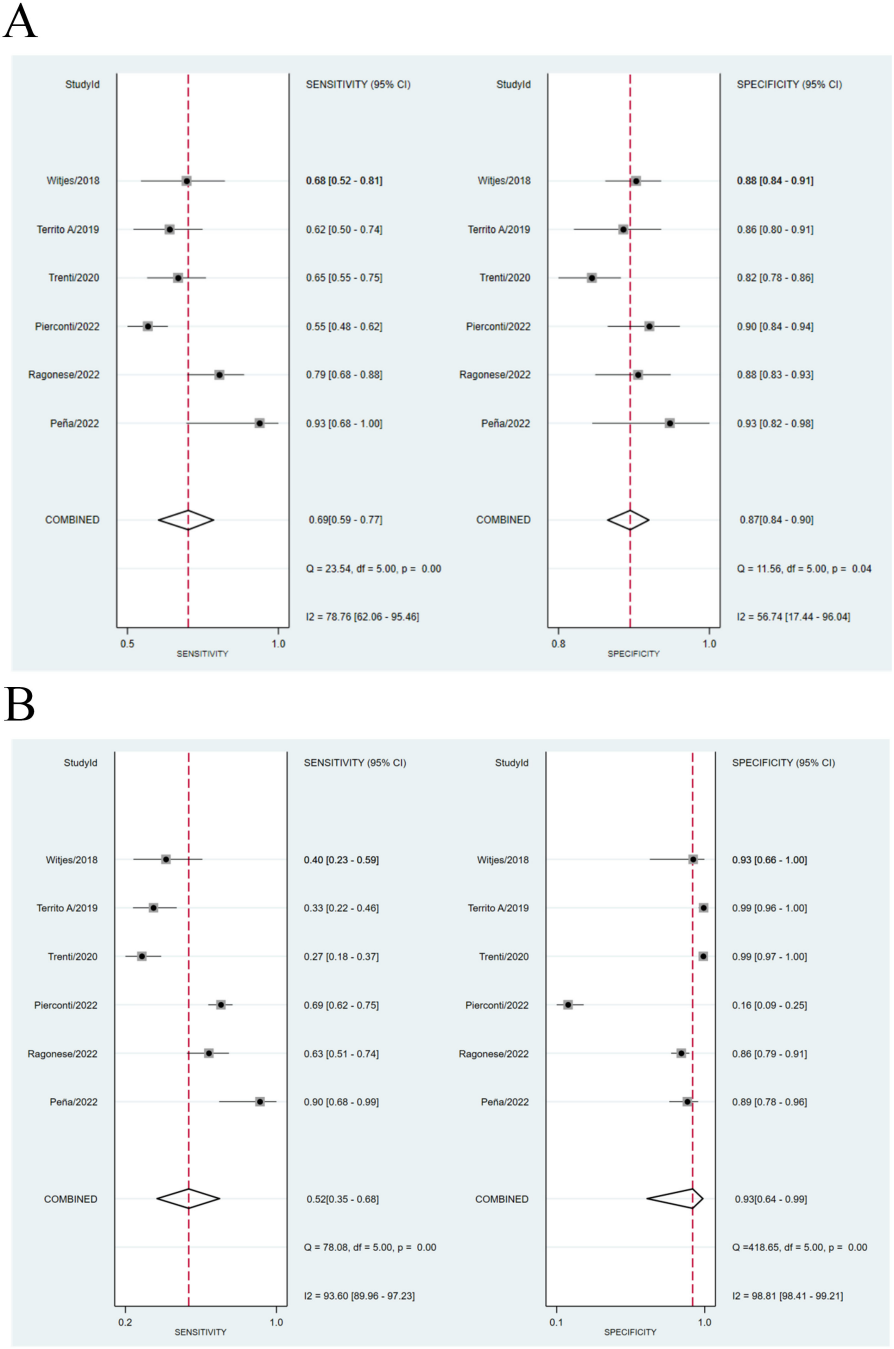
**(A)** Comparison of sensitivity between Urine Methylation Test (left) and cytology (right) of NMIBC. **(B)** Comparison of specificity between Urine Methylation Test (left) and cytology (right) of NMIBC.

**Figure 3:**
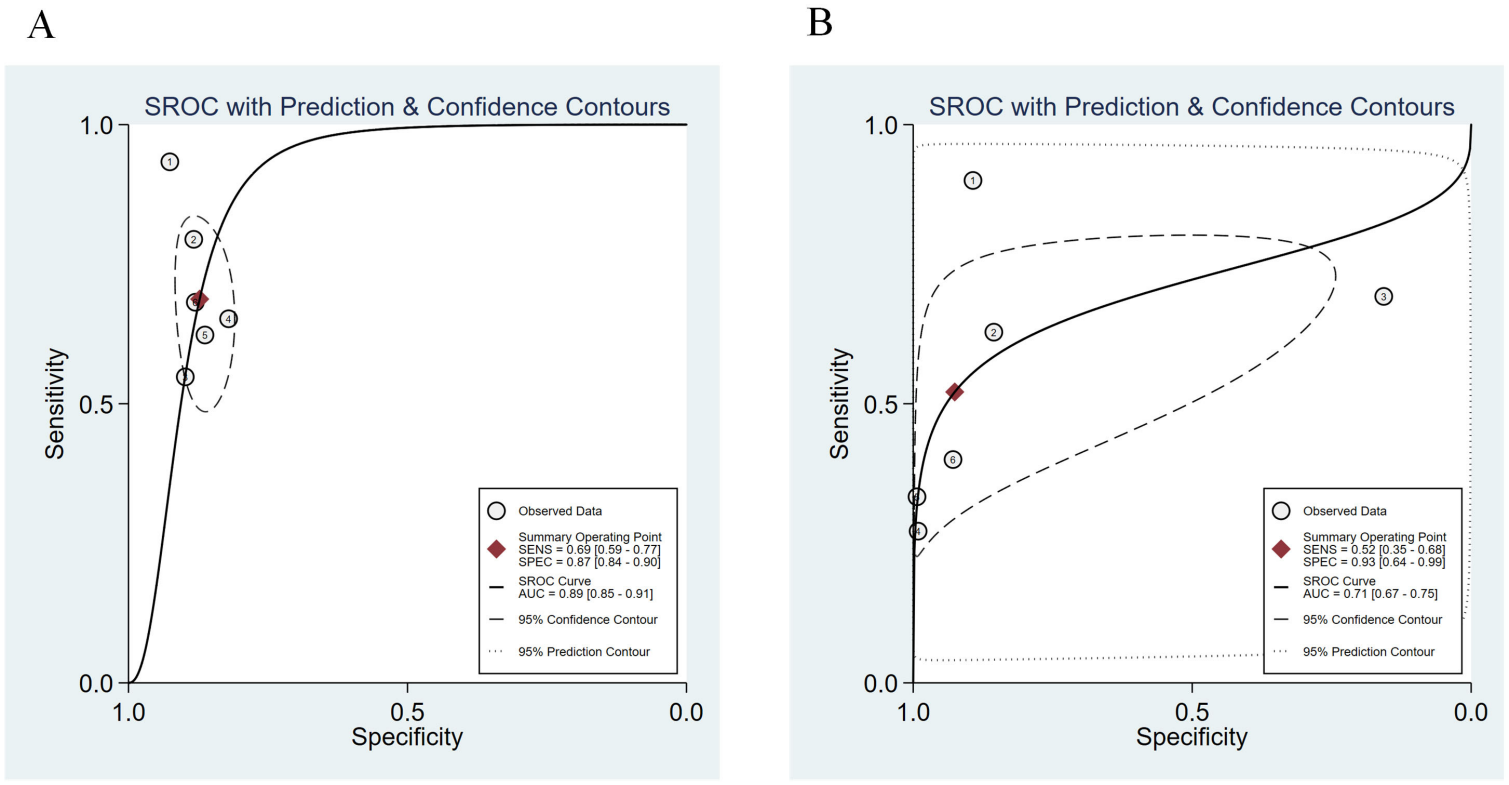
The summary receiver operating characteristic (SROC) curve of urine methylation test **(A)** and cytology **(B)** in the diagnosis of NMIBC.

We compared the sensitivity and specificity of the Urine Methylation Test with the cytology examination (**Figure 2**). In the urine methylation test, due to high heterogeneity (I^2^>50), the sensitivity was 0.69 (95% confidence interval (CI) 0.59 to 0.77, I^2^ = 77%) in the random effects model, while the pooled specificity was 0.87 (95% CI 0.84-0.90, I^2^ = 57%). In the cytology examination, the sensitivity was 0.52 (95% CI 0.35 to 0.68, I^2^ = 94%) in the random effects model, while the specificity was 0.93 (95% CI 0.64-0.99, I^2^ = 99%). The area under curve (AUC) for receiver operating characteristic (ROC) of urine methylation test was 0.89 (95% CI 0.85-0.91, I^2^ = 55%), and AUC was 0.71 (95% CI 0.67-0.75, I^2^ = 62%) for the cytology examination (**Figure 3**). Furthermore, as shown by Pena, Ragonese, Trenti, and Territo, urine methylation test has a higher sensitivity in detecting high-grade NMIBC, as indicated in **Table 3**.

### Research quality assessment

**Figure 4:**
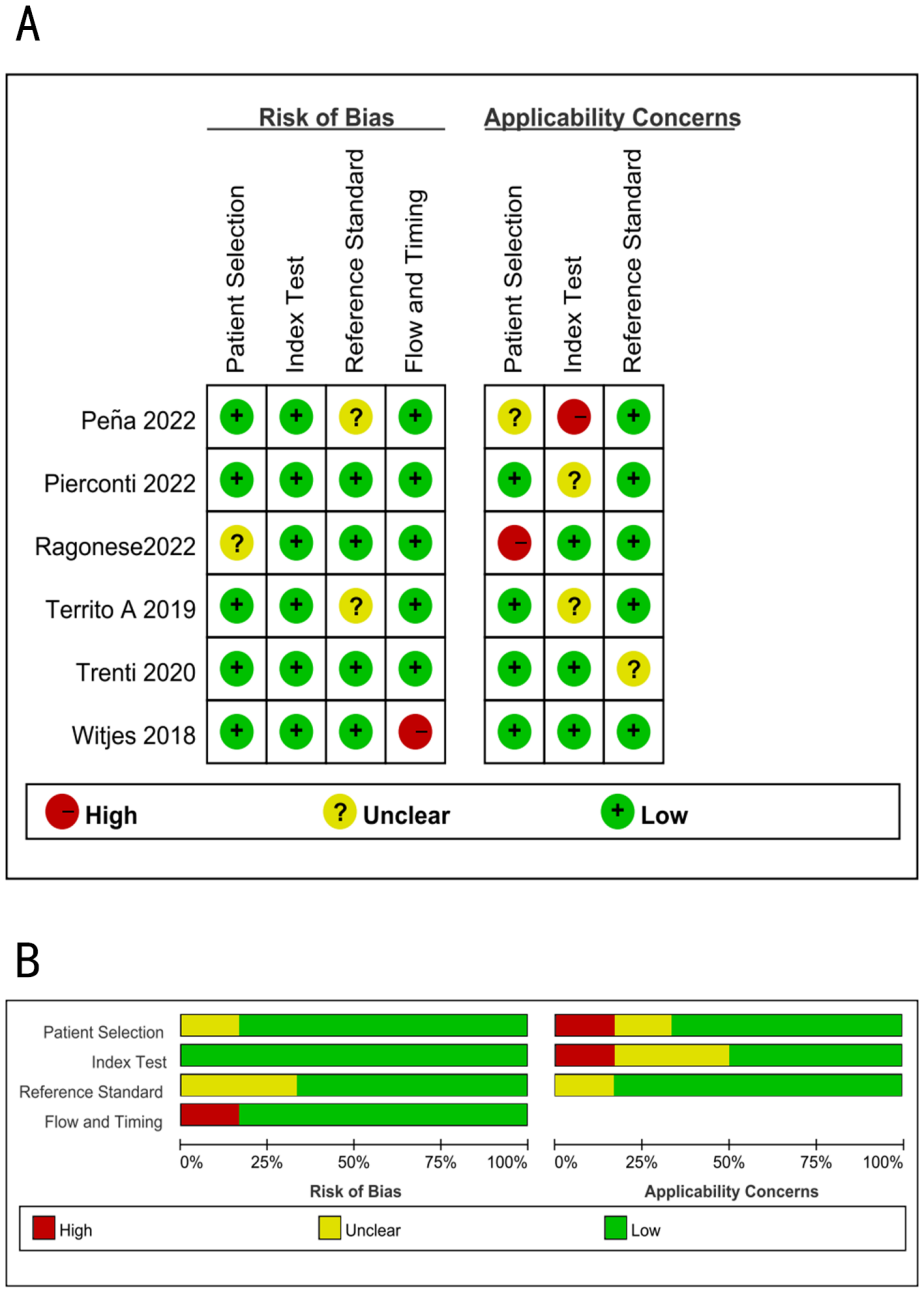
Summary of methodological quality of all studies using the QUADAS-2; **(A)** Risk of bias graph; **(B)** Risk of bias summary.

**Figure 4** illustrates the methodological quality of six studies. A study (22) examined the potential bias in patient selection, which was not clearly defined. Two studies (17, 20) reported the possibility for bias due to unclear reference criteria. One study (21) had a significant probability of bias in flow and timing. One study (22) had a substantial risk of bias, while another (17) had an unclear risk of bias in patient selection. One study (17) had a substantial risk of bias, whereas two studies (19, 23) had an uncertain risk of bias in the index test. One study (23) had an unclear risk of bias in reference standards.

## Discussion

DNA methylation is a fundamental epigenetic process that has a significant influence for gene expression and imprinting. It modifies genes without affecting their DNA sequence and performs crucial activities such as protecting against viral sequences, suppressing recombination, and facilitating the assembly of heterochromatin (24). Previously, DNA methylation research focused on the regulation of epigenetic genes in the central nervous system, as well as potential therapeutic targets for the treatment of neuropsychiatric diseases (25). Previous study has found that DNA methylation was associated with tumorigenesis and progression, with tumor suppressor genes often being hypermethylated and oncogenes being hypomethylated (26). As research has progressed, the use of DNA methylation in cancer screening and follow-up has gradually gained attention, especially in NMIBC.

Patients with NMIBC frequently experience multiple relapses throughout their lives, and the disease may progress in some cases. This illness presents severe challenges for patients and healthcare systems, resulting in a substantial burden (27). The statistical data reported that 1% to 45% of NMIBC can progress to a muscle-invasive tumor form within 5 years. The risk of recurrence and progression increases with the following factors: stage, grade of malignancy, size and number of lesions, and presence of carcinoma *in situ* (28). The quality of follow-up directly affects the mortality of NMIBC. Urine cytology, although being the most widely used non-invasive diagnostic test for BC, has a low sensitivity. As a result, cystoscopy and biopsy remain to be standard diagnostic methods, despite the discomfort and psychological stress they cause patients.

In recent studies, researchers investigated novel urinary tract tests that depend on identifying DNA alteration in urothelial cells. These tests aim to overcome the limitations of traditional BC screening methods and have the potential to decrease the need for cystoscopies in patients with low-grade tumors who are under active surveillance (29, 30). According to new research (18), urine methylation test performs well in the diagnosis of NMIBC. According to the meta-analysis, urine methylation test had higher sensitivity than cytology (0.69 vs. 0.52). In each included study, different investigators also found the same conclusions regarding the effectiveness of the two methods in diagnosing NMIBC. Furthermore, according to Pena, Ragonese, Trenti, and Territo et al, urine methylation test has a higher sensitivity in the diagnosis of high-grade NMIBC.

To better show the linear correlation between sensitivity and specificity, the researchers constructed the ROC curve based on the odds ratio (OR) weight from each study, referred to as the SROC curve. The SROC curve is unaffected by variations in the diagnostic threshold and may directly reflect the efficacy of diagnostic test using the Fig and Area (30). The AUC of the ROC curve represents the accuracy of diagnostic test. After analysis, the SROC curve of urine methylation test is compatible with the Fig that should be determined as the examination technique with high diagnostic value, with an AUC value of 0.89, which is larger than that of cytology (0.71). It should be mentioned that the specificity of urine methylation test is lower than that of the cytology test, which is a limitation of this technique.

It is critical to understand the limitations of this study. The papers included in the analysis had several limitations in terms of study design, patient characteristics, pathologist expertise, and incomplete data. In addition, selective and subjective factors should be considered. Therefore, the results should be interpreted with caution. The effectiveness of urine methylation test and cytology in the diagnosis of NMIBC must to be validated by investigations with larger sample size.

## Conclusions

Urine methylation test have proven to be high sensitivity and accuracy in monitoring patients with NMIBC after surgery. However, their specificity is lower than cytology. There are a limited number of studies on the role of urine methylation test in monitoring NMIBC. More research is needed to conduct a meta-analysis.

## Data Availability

The raw data supporting the conclusions of this article will be made available by the authors, without undue reservation.
